# Classifying Chewing and Rumination in Dairy Cows Using Sound Signals and Machine Learning

**DOI:** 10.3390/ani13182874

**Published:** 2023-09-10

**Authors:** Saman Abdanan Mehdizadeh, Mohsen Sari, Hadi Orak, Danilo Florentino Pereira, Irenilza de Alencar Nääs

**Affiliations:** 1 Department of Mechanics of Biosystems Engineering, Faculty of Agricultural Engineering and Rural Development, Agricultural Sciences and Natural Resources University of Khuzestan, Ahvaz 63417-73637, Iran; hadiorak@yahoo.com; 2 Department of Animal Sciences, Faculty of Animal Sciences and Food Technology, Agricultural Sciences and Natural Resources University of Khuzestan, Ahvaz 63417-73637, Iran; m.sari@asnrukh.ac.ir; 3 Department of Management, Development and Technology, School of Science and Engineering, Sao Paulo State University, Tupã 17602-496, SP, Brazil; danilo.florentino@unesp.br; 4 Graduate Program in Production Engineering, Paulista University—UNIP, São Paulo 04026-002, SP, Brazil; irenilza@agr.unicamp.br

**Keywords:** dairy farming industry, rumination patterns, sound signals analysis, textural features, machine learning, genetic algorithm

## Abstract

**Simple Summary:**

This article aims to investigate the nutritional behavior of dairy cattle, aiming to comprehend their dietary requirements and eating habits. In this regard, an effort has been made to scrutinize dietary patterns by analyzing sound recordings captured from the cows’ jaws during the chewing process. The paper outlines the methodology for developing various models to discern nutritional patterns in dairy cattle, employing six well-known classifiers. Understanding nutritional behavior and dietary patterns in dairy cattle is crucial for livestock managers and animal welfare. By comprehending the dietary requirements and eating habits of dairy cattle, managers can ensure that the cows are receiving the appropriate nutrients to maintain their health and productivity. This information can also help managers identify any potential health issues or deficiencies in the cows’ diets, allowing for early intervention and prevention of further health problems. Additionally, understanding the nutritional behavior of dairy cattle can lead to more efficient feeding practices, reducing waste and costs associated with overfeeding or underfeeding. Ultimately, establishing an appropriate pattern for evaluating the nutrition of dairy cattle can serve as a valuable guide for livestock managers to ensure the well-being and welfare of the cows while also improving the overall productivity and profitability of the dairy farm.

**Abstract:**

This research paper introduces a novel methodology for classifying jaw movements in dairy cattle into four distinct categories: bites, exclusive chews, chew-bite combinations, and exclusive sorting, under conditions of tall and short particle sizes in wheat straw and Alfalfa hay feeding. Sound signals were recorded and transformed into images using a short-time Fourier transform. A total of 31 texture features were extracted using the gray level co-occurrence matrix, spatial gray level dependence method, gray level run length method, and gray level difference method. Genetic Algorithm (GA) was applied to the data to select the most important features. Six distinct classifiers were employed to classify the jaw movements. The total precision found was 91.62%, 94.48%, 95.9%, 92.8%, 94.18%, and 89.62% for Naive Bayes, k-nearest neighbor, support vector machine, decision tree, multi-layer perceptron, and k-means clustering, respectively. The results of this study provide valuable insights into the nutritional behavior and dietary patterns of dairy cattle. The understanding of how cows consume different types of feed and the identification of any potential health issues or deficiencies in their diets are enhanced by the accurate classification of jaw movements. This information can be used to improve feeding practices, reduce waste, and ensure the well-being and productivity of the cows. The methodology introduced in this study can serve as a valuable tool for livestock managers to evaluate the nutrition of their dairy cattle and make informed decisions about their feeding practices.

## 1. Introduction

The dairy farming industry is crucial in providing essential nutrients to the human population, including high-quality protein, energy, vitamins, and minerals. As the annual milk production of cows increases, their nutritional requirements also rise throughout the production cycle. The simultaneous demands of milk production and reproduction in dairy cows necessitate careful nutritional management to achieve optimal production and consecutive breeding. Livestock farmers must provide for their cows’ nutritional needs at each production stage based on their output level. Overfeeding can cause obesity, decreased milk production, infertility, and metabolic disorders such as ketosis and fatty liver [1]. Farmers must balance increasing milk production with providing nutrient-rich feeds to meet the cows’ needs.

On the other hand, failure to meet nutritional needs can result in weight loss, decreased production, infertility, gestation, and delivery problems. It is crucial to ensure that dairy cows receive balanced and adequate nutrition to prevent these issues and promote their overall health and productivity [2]. Advancements in dairy technology have increased the demand for dairy products. This increased demand has eliminated the need for farmers to seek buyers actively and increased the number of cattle per farm [3]. As a result, farmers have less time to spend on each cow. Precision dairy farming technology has gained popularity among farmers and the industry due to its ability to increase efficiency and profitability while reducing workload [4,5].

As the scale of dairy operations expands, the task of monitoring cows grows more intricate and requires enhanced management skills. Adopting automation and sensor systems, known as precision technology, allows farmers to reduce labor requirements, improve resource efficiency, and improve cows’ health and welfare [6,7,8].

Daily rumination patterns are widely recognized as crucial indicators of individual cows’ health and productivity. Measuring these parameters can provide valuable insights into nutritional physiology for research purposes [9]. In dairy farming, early detection of physiological and behavioral changes or abnormalities is essential to minimize losses in milk yield and health costs. Automated monitoring of feeding behavior and activity can serve as both a valuable research tool for scientists and an early warning system for farmers [10]. Currently, various technologies are available for monitoring different aspects of animal behavior and health, including developmental activities [11,12] and feeding behavior [13,14,15]. However, many of these technologies have limitations, such as being able to measure only one type of behavior or being less practical for everyday use. Some systems may require specialized training or be challenging to install and maintain. Consequently, it is imperative to engage in further investigation and advancement in this domain to generate more efficient and accessible mechanisms for animal surveillance and administration.

Cow audio recording while eating is a relatively new field of research that has gained significant attention in recent years. By utilizing sound sensors to recognize cow chewing behavior, researchers can monitor these animals’ health and physiological status [16]. This technology provides numerous benefits to farmers, allowing them to monitor their cows’ health and physiological status more efficiently and accurately. By recognizing chewing behavior, farmers can judge the health status of their cows and make informed decisions about their care. Additionally, cow audio recording can be integrated with other technologies to develop real-time monitoring systems for livestock [17]. One study employed a neural network to recognize chewing behavior by inputting many labeled ruminating and eating audio data into the network [18]. Another research proposed a semi-automatic tool for labeling monophonic cow sound events, allowing users to quickly designate the temporal onset and offset points for audio events or make new annotations [19]. One study monitored and assessed ingestive chewing sounds to predict herbage intake rate in grazing cattle. The study found that by utilizing sound sensors to recognize cow chewing behavior, researchers could accurately predict herbage intake rates in grazing cattle [20]. Another study developed a web-based monitoring and recording system based on artificial intelligence analysis to classify cattle sounds. The deep learning classification model is a convolutional neural network (CNN) model that takes voice information converted to Mel-frequency cepstral coefficients (MFCCs) as input. The developed model was applied to cattle sound data from an on-site monitoring system through sensors, achieving a final accuracy of 81.96% [21].

The hypothesis of this study posits that it is possible to distinguish between chewing and ruminating states in dairy cattle based on the analysis of sound signals captured by microphones installed near the cows’ mandibles. Therefore, the present research study explores cows’ feeding behavior to comprehend their nutritional needs and feeding habits. The study examines the audio recordings of cows during their feeding process to evaluate the frequency and patterns of their chewing and rumination. Through this analysis of cow behavior, the study aims to enhance the understanding of animal health and welfare and facilitate the development of more efficacious feeding practices for cattle farmers and ranchers. The outcomes of this study are anticipated to be pertinent for researchers and industry professionals.

## 2. Materials and Methods

**Figure 1:**
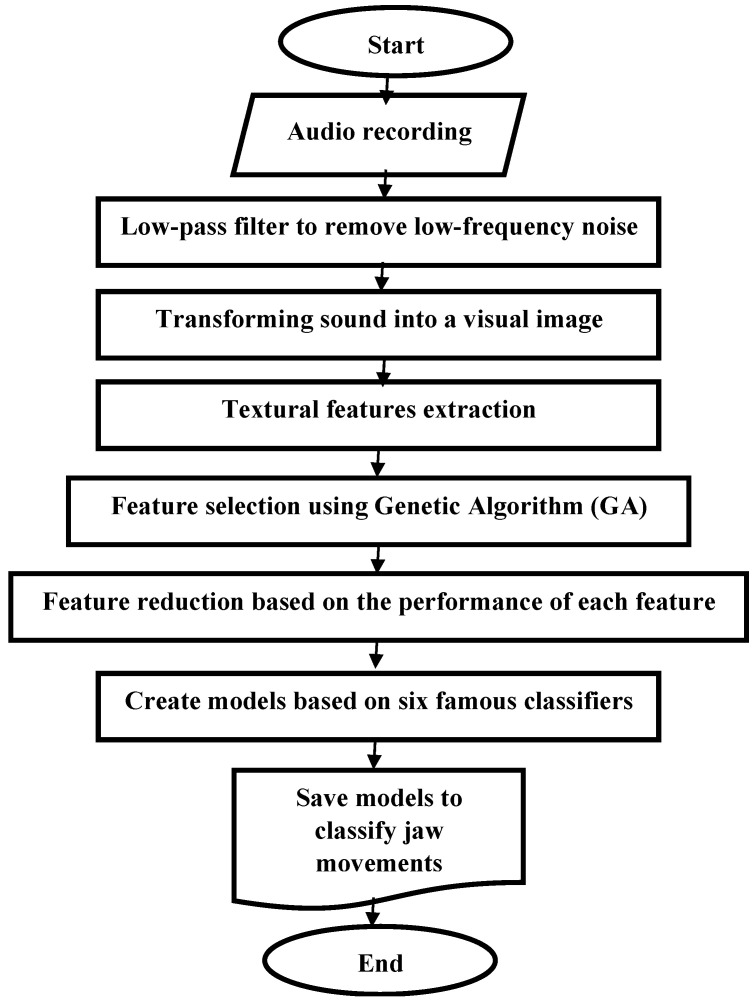
The flowchart of the pattern recognition algorithm for JM classification.

The flowchart of the proposed pattern recognition algorithm is depicted in Figure 1. The methodology comprises several stages that facilitate the processing, description, and analysis of sound signals in a generic manner. These stages include recording audio sound, converting audio to images, extracting textural features, selecting features, and performing classification. The algorithm can distinguish cows’ jaw movement (JM) events into four distinct classes while consuming two different particle size distributions of wheat straw and Alfalfa hay. These classes include exclusive bites (B—bites taken during feeding), exclusive chews (C—chews related solely to ingestion), chew-bite combination (CB—compound chew-bite taken during feeding), and exclusive sorting (S—jaw movement that creates holes to separate feed particles). The chew bites intelligent algorithm using heuristic features is capable of real-time pattern recognition, enabling it to detect and categorize masticatory sounds produced by ruminants into four JM classes [1]. Further details on these stages will be provided in the subsequent sections.

### 2.1. Data Collection and Sound Recording

In previous studies, researchers used strategically positioned microphones on animals’ foreheads to explore and analyze their chewing sounds. These investigations were primarily centered on cattle [22] and sheep [23]. The findings revealed that these microphones effectively differentiate between feeding and rumination behaviors [22,23]. The current research also employed a similar methodology to collect information in line with this approach. A microphone was carefully attached to the forehead of the cattle, and a specially designed harness was utilized to hold the microphone securely in position to ensure stability and proper placement. Ten lactating Holstein cows, aged between 4 and 6 years and weighing approximately 603 ± 31.4 kg, were selected for the study. These cows had been previously acclimated and underwent training before the research commenced. The dataset comprises 60 audio recordings of these cows consuming food of varying particle sizes. Particle size distribution was determined using a Penn State Particle Separator outfitted with three sieves (19, 8, and 1.18 mm) and a bottom pan. Samples were categorized as either tall (≥19 mm) or short (>1.18 to <8 mm). The proportion of tall and short particle sizes in wheat straw and Alfalfa hay on the experimental farm typically ranged from approximately 2 to 4 percent and 50 to 55 percent (based on dry matter), respectively. The audio recordings ranged from 100 to 300 s and captured 3508 digestive behaviors, including biting, chewing, chewing-biting, and sorting. In addition to the audio recordings, videos were also obtained simultaneously to provide visual confirmation and labeling of the observed behaviors, with the assistance of two expert individuals.

### 2.2. Signal Pre-Possessing and Short-Time Fourier Transformation

The signal conditioning stage involves using an adaptive low-pass filter to condition and filter the sound signal, improving its signal-to-noise ratio (SNR). After the signal conditioning stage, the sound signals were processed using the short-time Fourier transform (STFT) algorithm, which involved dividing the signal into small segments and computing the Fourier transform of each segment. This allowed the frequency content of the sound signal over time to be analyzed. The STFT algorithm was implemented using self-compiled code in Matlab R2021b. A sliding window approach was specifically used to divide the sound signal into overlapping segments of length 2048 samples with a hop size of 1024 samples. Each segment was subjected to a Hamming window function to reduce spectral leakage. The resulting spectrogram was then normalized by taking the logarithm of the magnitude of the STFT and scaling it between 0 and 1. This produced a two-dimensional time-frequency image that represented the sound signal. The STFT of a signal *f*(*t*) is obtained through a convolution integral [24] using Equation (1).

(1) $$S\left(\tau ,f\right)=\int_{-\infty }^{\infty }f\left(t\right)\frac{\left|f\right|}{\sqrt{2\pi }}e^{\left[\frac{-f^{2}{\left(\tau -t\right)}^{2}}{2}\right]}e^{-2\pi ft}dt$$

where *S*(*τ*, *f*) is the *S* transform of *f*(*t*), *f* is the frequency, *t* is the time, and *τ* controls the position of the Gauss window function on the time axis.

**Figure 2:**
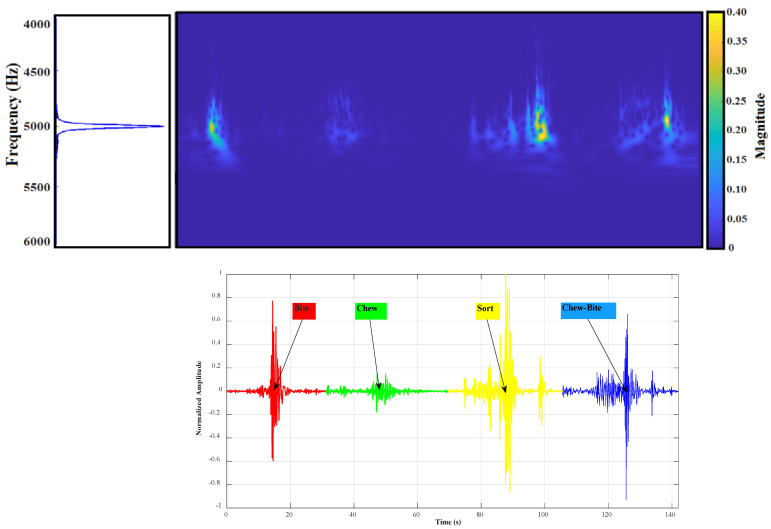
Short-time Fourier transform of four different JM events.

Figure 2 demonstrates an example of transforming the sound produced by cow JM events of four classes into a visual image using the STFT algorithm. The resulting images provided a representation of the sound signals that could be used for further analysis and classification.

### 2.3. Feature Extraction Using the Spectrum of the Sound Signal

#### 2.3.1. Gray Level Co-Occurrence Matrix

A Gray level co-occurrence matrix (GLCM) is a mathematical representation of the relationship between different gray levels in an image. It is structured as a square matrix with the number of rows and columns equal to the number of gray levels. Each element in the matrix, denoted as *S*(*i*,*j*|∆*x*, ∆*y*), represents the relative frequency of occurrence of two pixels with intensities ‘*i*’ and ‘*j*’ within a certain distance (∆*x*, ∆*y*) from each other in the image. Similarly, another element in the matrix, denoted as *S*(*i*,*j*|*d*,*θ*), contains second-order statistical probability values for the changes between gray levels ‘*i*’ and ‘*j*’ at a specific displacement distance ‘*d*’ and angle ‘*θ*’. The GLCM provides valuable information about the spatial relationships between different gray levels, helping to capture texture patterns and structural information in the image. However, due to the large number of possible gray levels, constructing a complete GLCM for each combination of (∆*x*, ∆*y*) or (*d*, *θ*) can result in substantial memory requirements. As a result, to reduce dimensionality and manage computational complexity, the number of gray levels is often reduced before computing the GLCM. This reduction helps in efficiently extracting relevant texture features while mitigating the sensitivity of GLCM to the size of the texture samples used for estimation [25]. After the GLCM was formed, the ensuing features were extracted using Equations (2)–(12).
Contrast$Con=\sum_{i,j=0}^{N-1}S_{i,j}{\left(i-j\right)}^{2}$(2)Correlation$Cor=\sum \frac{\left(i-\mu \right)\left(j-\mu \right)S_{i,j}}{\sigma^{2}}$(3)Autocorrelation$ACor=\sum_{i=1}^{NG}\sum_{j=1}^{NG}ijS_{0}\left(i,j\right)$(4)Joint Average$JA=\sum_{i=1}^{NG}\sum_{j=1}^{NG}S_{0}\left(i,j\right)i$(5)Cluster Prominence$CP=\sum_{i=1}^{NG}\sum_{j=1}^{NG}{\left(i+j-\mu_{x}-\mu_{y}\right)}^{4}S_{0}\left(i,j\right)$(6)Cluster Shade$CS=\sum_{i=1}^{NG}\sum_{j=1}^{NG}{\left(i+j-\mu_{x}-\mu_{y}\right)}^{3}S_{0}\left(i,j\right)$(7)Cluster Tendency$CT=\sum_{i=1}^{NG}\sum_{j=1}^{NG}{\left(i+j-\mu_{x}-\mu_{y}\right)}^{2}S_{0}\left(i,j\right)$(8)Joint Energy$JE=\sum_{i=1}^{NG}\sum_{j=1}^{NG}S_{0}{\left(i,j\right)}^{2}$(9)Joint Entropy$JEnt=-\sum_{i=1}^{NG}\sum_{j=1}^{NG}S_{0}\left(i,j\right)log_{2}(S_{0}\left(i,j\right)+\varepsilon )$(10)Inverse Difference Moment Normalized$IDMN=\sum_{i=1}^{NG}\sum_{j=1}^{NG}\frac{S_{0}\left(i,j\right)}{1+\left(\frac{|i-j{|}^{2}}{NG^{2}}\right)}$(11)Inverse Difference$ID=\sum_{i=1}^{NG}\sum_{j=1}^{NG}\frac{S_{0}\left(i,j\right)}{1+\left|i-j\right|}$(12)

#### 2.3.2. The Spatial Gray Level Dependence Method

The spatial gray level dependence method (SGLDM) is a technique that relies on estimating second-order joint conditional probability density functions, denoted as *S*(*i*,*j*|*d*,*θ*), where 0 represents angles in increments of 45 degrees (0°, 45°, 90°, 135°, 180°, 225°, 270°, 315°). These probability density functions indicate the likelihood of transitioning from gray level *i* to gray level *j*, considering the inters ample spacing denoted as d and the specified angle denoted as 0. The estimated values for these probability density functions are denoted as *S*(*i*,*j*|*d*,*θ*), where 0 takes the values of 0°, 45°, 90°, 135°, 225°, 270°, and 315°. This estimation process forms the basis of the spatial gray level dependence method (SGLDM) [26,27,28,29]. Upon the creation of the SGLDM, a set of seven distinct features is derived following Equations (13)–(19).
Energy$E\left(S_{0}\left(d\right)\right)=\sum_{i=0}^{NG-1}\sum_{j=0}^{NG-1}{\left[S_{0}(i,j|d)\right]}^{2}$(13)Entropy$H\left(S_{0}\left(d\right)\right)=-\sum_{i=0}^{NG-1}\sum_{j=0}^{NG-1}S_{0}(i,j|d)log\;S_{0}(i,j|d)$(14)Correlation$C\left(S_{0}\left(d\right)\right)=\frac{\sum_{i=0}^{NG-1}\sum_{j=0}^{NG-1}(i-\mu_{x})\left(j-\mu_{y}\right)\;S_{0}(i,j|d)}{\sigma_{x}\sigma_{y}}$(15)Local Homogeneity$L\left(S_{0}\left(d\right)\right)=-\sum_{i=0}^{NG-1}\sum_{j=0}^{NG-1}\frac{1}{1+{\left(i-j\right)}^{2}}\;S_{0}(i,j|d)$(16)Inertia$I\left(S_{0}\left(d\right)\right)=-\sum_{i=0}^{NG-1}\sum_{j=0}^{NG-1}{\left(i-j\right)}^{2\;}\;S_{0}(i,j|d)$(17)Mean$Mean=\sum_{i}\sum_{j}\;S_{0}\left(i,j\right)$(18)Variance$Var=\sum_{i}\sum_{j}{\left(i-mean\right)}^{2}\;S_{0}\left(i,j\right)$(19)

#### 2.3.3. The Gray Level Run Length Method

Referred to as the GLRLM, it utilizes a technique of calculating the occurrences of gray-level runs of different lengths. A gray level run refers to a consecutive set of pixels in an image that shares the same gray level value. The length of a run is determined by the number of pixels within that run. The gray level run length matrix, denoted as *S*(*i*, *θ*):

(20) $$R\left(\theta \right)=\left[S_{0}\left(i,j|\theta \right)\right]$$

This is employed to indicate the estimated frequency of runs of length *i* for gray level *i* in a specific direction *θ*. For each image, four gray level run length matrices *R*(*θ*), where *θ* represents the angles 0°, 45°, 90°, and 135°, are computed. The matrices yield a compilation of nine distinct features for each matrix, ascertained through using Equations (21)–(29).
Short Run Emphasis$SRE=\frac{\sum_{i=1}^{NG}\sum_{j=1}^{Nr}\frac{\;S_{0}(i,j|\theta )}{j^{2}}}{N_{Z}\left(\theta \right)}$(21)Long Run Emphasis$LRE=\frac{\sum_{i=1}^{NG}\sum_{j=1}^{Nr}S_{0}(i,j|\theta )j^{2}}{N_{Z}\left(\theta \right)}$(22)Gray Level Non-Uniformity$GLNU=\frac{\sum_{i=1}^{NG}{\left(\sum_{j=1}^{Nr}S_{0}(i,j|\theta )\right)}^{2}}{N_{Z}\left(\theta \right)}$(23)Run Length Non-Uniformity$RLNU=\frac{\sum_{j=1}^{Nr}{\left(\sum_{i=1}^{NG}S_{0}(i,j|\theta )\right)}^{2}}{N_{Z}\left(\theta \right)}$. (24)Run percentage$Rp=\frac{N_{Z}\left(\theta \right)}{N_{S}}$(25)Low Gray Level Run Emphasis$LGLRE=\frac{\sum_{i=1}^{NG}\sum_{j=1}^{Nr}\frac{\;S_{0}(i,j|\theta )}{i^{2}}}{N_{Z}\left(\theta \right)}$(26)High Gray Level Run Emphasis$HGLRE=\frac{\sum_{i=1}^{NG}\sum_{j=1}^{Nr}S_{0}(i,j|\theta )i^{2}}{N_{Z}\left(\theta \right)}$(27)Gray Level Variance$GLV=\sum_{i=1}^{NG}\sum_{j=1}^{Nr}S_{0}(i,j|\theta ){\left(i-\mu \right)}^{2}$(28)Run Variance$RV=\sum_{i=1}^{NG}\sum_{j=1}^{Nr}S_{0}(i,j|\theta ){\left(j-\mu \right)}^{2}$(29)

#### 2.3.4. The Gray Level Difference Method

In order to explain the gray level difference method (GLDM) [30] using alternative wording, we can start by considering a digital image represented by the function *g*(*n*, *m*), where ‘*n*’ and ‘*m*’ denote the coordinates of the pixels. The GLDM calculates the difference in gray levels between two pixels based on a given displacement vector, denoted as *δ* = (Δ*n*, Δ*m*), where Δ*n* and Δ*m* are integers. This difference is defined as *gδ*(*n*, *m*) = |*g*(*n*, *m*) − *g*(*n* + Δ*n*, *m* + Δ*m*)|. To estimate the probability density function associated with the potential values of *gδ*, let us denote it as *f*′(*gδ*). This function provides information about the likelihood of encountering different values of *gδ* within the image.

(30) $$f^{\prime }\left(i|\delta \right)=P\left(g_{\delta }\left(n,m\right)=i\right)$$

In this examination, four potential representations were examined, namely (0, *d*), (−*d*, *d*), (*d*, 0), and (−*d*, −*d*), where d denotes the inter-sample spacing distance. The density functions for the gray level difference are denoted as *f*′(0|*δ*). These density functions are subjected to Equations (31)–(35) to derive five distinct texture features as follows:
Dependence Entropy$DE=-\sum_{i=1}^{NG}\sum_{j=1}^{NR}f^{\prime }(i|\delta )log_{2}[f^{\prime }\left(i|\delta \right)+\varepsilon ]$(31)Small Dependence Low Gray Level Emphasis$SDLGLE=\frac{\sum_{i=1}^{NG}\sum_{j=1}^{Nr}\frac{\;f^{\prime }\left(i|\delta \right)}{i^{2}j^{2}}}{N_{Z}}$(32)Small Dependence High Gray Level Emphasis$SDHGLE=\frac{\sum_{i=1}^{NG}\sum_{j=1}^{Nr}\frac{\;f^{\prime }\left(i|\delta \right)}{i^{2}}}{N_{Z}}$(33)Small Dependence Emphasis$SDE=\frac{\sum_{i=1}^{NG}\sum_{j=1}^{Nr}\frac{\;f^{\prime }\left(i|\delta \right)}{i^{2}}}{N_{Z}}$(34)Large Dependence Emphasis$LDE=\frac{\sum_{i=1}^{NG}\sum_{j=1}^{Nr}f^{\prime }{\left(i|\delta \right)}^{2}}{N_{Z}}$(35)

### 2.4. Feature Selection Using Genetic Algorithm

Image recognition systems can experience reduced classification accuracy due to redundant or irrelevant features within high-dimensional feature sets. Various features were investigated to address this issue, and the most pertinent subset that maximizes the accuracy was selected. Feature selection, also referred to as variable selection, is a process in machine learning and statistics that aims to find a subset of relevant features that are optimal for constructing machine models. Several techniques exist for feature selection, such as principal component analysis (PCA), particle swarm optimization (PSO), and genetic algorithm (GA), as documented in [31,32].

Genetic algorithms (GA) are a class of optimization techniques that employ a population-based search heuristic inspired by natural evolution mechanisms ([33,34,35]). In a GA, a population of chromosomes (solution candidates) is iteratively modified by applying genetic operators such as crossover and mutation. This research utilized a genetic algorithm to reduce the number of features. Compared to other feature selection techniques such as PCA and PSO, GA has the advantage of being able to handle nonlinear relationships between features and can search for global optima rather than getting stuck in local optima. GA is also able to handle large feature sets without significantly increasing computational time. By utilizing the GA for feature selection, the number of features in the dataset was successfully reduced while simultaneously maintaining or even enhancing the classification accuracy. This not only improves the efficiency of the classification models by reducing computational time and memory requirements, but it also improves the interpretability of the models by focusing on the most relevant features. The criteria used for feature selection were based on the concepts presented in Table 1.

### 2.5. Classification of Mastication Behavior

Managing systems requires accurate measurement of animal feeding behavior to help monitor their health and welfare and improve resource management. This is achievable through a precise understanding of the feeding behavior of ruminant cows. The investigated feeding behaviors include biting and chewing, chewing-biting, and separation. Therefore, achieving this goal in the first step requires using an effective classifier and proper classification of the mentioned behaviors in the second step.

In order to classify different states of the food digestion process (jaw movements), six well-known classifiers were used: (i) naive Bayes, (ii) k-nearest neighbor, (iii) support vector machine, (iv) decision tree, (v) multi-layer perceptron and (vi) k-means clustering. In order to train the classifiers, two-thirds of the samples were utilized, while one-third were employed for testing purposes.

#### 2.5.1. Naive Bayes Classifier

Naive Bayes is a statistical learning algorithm that utilizes Bayesian networks [36] for probabilistic classification. During training, it estimates prior probabilities and conditional probabilities from the dataset. The prior probability for a specific class is calculated by dividing the count of training examples belonging to that class by the total number of examples. Conditional probabilities are determined based on the frequency distribution of feature *xi* within the training data for that particular class. It is capable of handling both binary and multi-class classification problems and exhibits efficiency and robustness when dealing with high-dimensional data. Nevertheless, its assumption of conditional independence among features may not always be valid, and its performance may be suboptimal when there are complex relationships between features and classes [37].

#### 2.5.2. K-Nearest Neighbor Classifier

K-nearest neighbor (KNN) is a simple yet robust algorithm that effectively handles complex classification problems. It considers multiple parameters, such as the number of nearest neighbors (denoted as *K*) to be considered during classification and the distance between features in the dataset, to determine the group to which a particular data point belongs. KNN is a nonparametric classifier that is well-suited for problems with irregular decision boundaries and can capture nonlinear relationships between features. It can also adapt effectively to new data, particularly when there are few features and many training examples. However, it has several disadvantages, including computational expense, particularly for large datasets and high dimensions, sensitivity to noise, outliers, and irrelevant features. In the proposed methodology, KNN was implemented with a *K* value 2 and utilized the cosine distance metric.

#### 2.5.3. Support Vector Machine Classifier

Support vector machine (SVM) employs a linear equation constructed from the training data to partition the dataset. The SVM algorithm operates in two steps: first, it maps nonlinear data from the input space to a feature space, and then it measures the similarity of feature vectors using a kernel function. SVM is known for handling large feature sets with high accuracy [38]. SVM is one of the most renowned methods for optimizing the expected solution [39,40]. Vapnik introduced SVM as a kernel-based machine learning model for classification and regression tasks. In recent years, SVM’s exceptional generalization capability, optimal solution, and discriminative power have attracted considerable attention from data mining, pattern recognition, and machine learning communities. SVM has proven to be a powerful tool for solving practical binary classification problems and has demonstrated superiority over other supervised learning methods [41,42,43]. However, it lacks probability estimates, sensitivity to the choice of kernel function and parameters, and has poor performance when the number of features is much greater than the number of samples or when classes are imbalanced. Owing to its solid theoretical foundations and excellent generalization capacity, SVMs have become one of the most widely adopted classification methods in recent years [44].

#### 2.5.4. Decision Tree Classifier

A decision tree (DT) is a graphical representation resembling a flowchart with rectangular nodes for internal decisions and oval nodes for results. It is widely employed due to its straightforward implementation and superior comprehensibility to alternative classification algorithms [45]. Decision tree construction is notably quick in comparison to other classification methods. Decision tree classifiers yield comparable, and occasionally superior, accuracy compared to alternative classification techniques. It possesses the capability to handle both numerical and categorical features, as well as missing values, is good at finding complex relationships in the data, and is useful when the data has a hierarchical structure. Depending on factors such as data volume, available memory space, and algorithm scalability, a decision tree algorithm can be executed sequentially or in a parallel fashion. Decision trees are also widely used in classification problems due to their faster training phases than neural networks. However, they exhibit a lack of flexibility with respect to model parameters, are prone to overfitting, are sensitive to small changes in the data, and may not perform optimally when there are numerous features or when the features are correlated [46,47].

#### 2.5.5. Multi-Layer Perceptron Classifier

The multi-layer perceptron (MLP) extends the feed-forward neural network, encompassing distinct layers, including the input, output, and hidden layers. The input layer receives input vectors intended for processing, while the output layer carries out tasks such as prediction and classification. Acting as the core computational component of the MLP, an arbitrary number of hidden layers are positioned between the input and output layers. Analogous to a feed-forward network, data propagates forward through the MLP, moving from the input to the output layer. Neurons within the MLP are trained using the backpropagation learning algorithm. The MLP, which was designed to classify four jaw movements, is capable of resolving misclassifications that are not linearly separable. This makes it suitable for problems that involve complex relationships between the features. Neural networks represent one of the most frequently employed techniques [48,49]. Neural networks find extensive application in various domains due to their universal approach. However, the design of a neural network for solving a particular problem requires the consideration of various factors, such as the learning algorithm, architecture, number of neurons per layer, number of layers, data representation, and others. Furthermore, neural networks are highly vulnerable to noise in the training data [50]. They also exhibit a tendency to overfit, which can be mitigated by regularization or dropout techniques [51].

#### 2.5.6. K-Means Clustering

The k-means algorithm, proposed by J.B. MacQueen, is a clustering algorithm that divides data into clusters [52]. It is commonly used in data mining and pattern recognition as an unsupervised learning technique. The algorithm aims to minimize the cluster performance index by utilizing the square error and error criteria. The algorithm seeks to find K divisions that satisfy a specific criterion to achieve an optimal outcome. A set of data points is chosen to represent the initial cluster centroids. Typically, the first K sample points are selected as the initial centroids. Subsequently, the remaining data points are assigned to their nearest centroid based on the minimum distance criterion, resulting in the initial classification. If the classification is deemed unreasonable, adjustments are made by recalculating the centroids of each cluster. This iterative process continues until a satisfactory classification is obtained. The k-means algorithm is based on dividing and offers the advantages of simplicity, efficiency, and speed in its execution. It is useful when there is no pre-defined class label for the data, and the goal is to discover the underlying structure or patterns in the data. However, this algorithm is sensitive to the initial selection of centroids, and different choices of initial samples may lead to divergent outcomes [53].

Additionally, the algorithm relies on a target function that employs the gradient method to find extrema. The gradient method searches for directions where energy decreases, which can pose challenges when the initial cluster centroids are not well-suited. In such cases, the algorithm may become trapped in local minimum points, affecting its overall performance [36]. The k-nearest-neighbor method is relatively straightforward. This technique exhibits slow performance when handling large input datasets and is highly sensitive to irrelevant parameters [54,55]. In this study, MATLAB 2022 software was employed to implement all classifiers.

## 3. Results

By transforming the sound signals emanating from feeding cows in different modes into visual representations, the resultant textural features of the images were regarded as potential attributes for employment in the classifier under investigation. Algorithms related to texture (GLCM, GLRLM, GLDM, and SGLDM) were used to classify jaw movements [56,57]. A total of 31 textural features were extracted from the images. However, only a few of these features were effective in classification performance. Therefore, in this study, feature selection consists of two main goals; the first goal involves eliminating irrelevant and non-informative features, and the second goal is to utilize sound and relevant features to enhance classification accuracy and speed.

**Figure 3:**
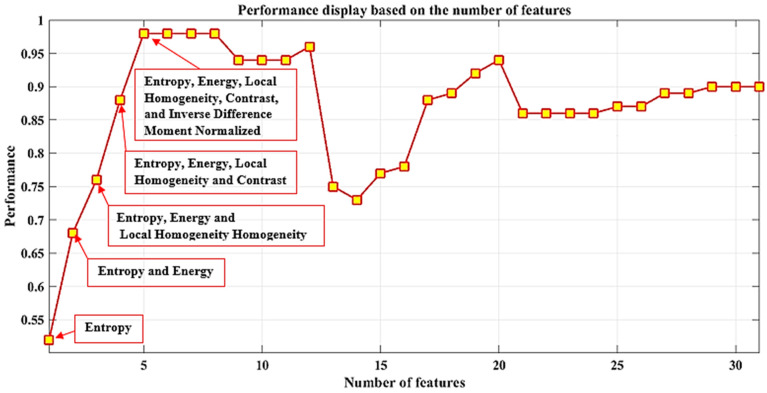
Performance of the relative importance of each feature.

A GA was employed for feature selection, which involves identifying the most pertinent and significant features from a large set of available features. The textural features extracted from images were entered into the GA to select the optimal features and enhance the classification performance. As illustrated in Figure 3, entropy, energy, local homogeneity, contrast, and inverse difference moment normalized were identified as the most impactful features, with performance percentages of 52%, 68%, 76%, 88%, and 98%, respectively.

In the process of analyzing jaw movements, it is anticipated that varying levels of sound intensity would be generated due to the significant impact of the lower jaw colliding with the upper jaw in each of the states—biting, chewing, chewing-biting, and sorting. This variation in sound intensity is represented as entropy, which measures the randomness or complexity of the image. A higher entropy value indicates more complexity and less uniformity. It is implied that an increase in sound intensity correspondingly increases the image energy, as the intensity of these generated sounds is directly proportional to the energy of the image. Higher energy values indicate more uniformity and less complexity. An uneven image matrix results from the sudden impacts of the jaw during biting and chewing-biting. This non-uniformity is expected to be reflected in the feature of local homogeneity, which measures the closeness of the distribution of elements in the GLCM to the GLCM diagonal. Images with a high degree of local homogeneity have pixel pairs with similar intensity. Image contrast is among the features selected by the genetic algorithm, as fluctuations in pixel intensity serve as an indicator thereof. Contrast measures the amount of local variations present in an image. During the chewing and sorting state, where sound intensity is relatively uniform, an image with uniform pixel intensity is anticipated. This is expected to align with a descriptor of grayscale color distribution uniformity in the image, represented as inverse difference moment normalized (IDMN). IDMN is a measure of the homogeneity of an image and gives higher values for homogeneous images.

Valuable insight into the characteristics of jaw movements is provided by these texture features and can be used to accurately classify different states. These features were identified as impactful because they capture essential characteristics of jaw movements. Entropy and energy can capture the complexity and uniformity of jaw movements, respectively. Local homogeneity can provide information about the similarity in jaw movements, while contrast can capture the amount of variation in these movements. Lastly, IDMN can provide a measure of how homogeneous these movements are.

Upon selecting the most prominent features, they were input into six classifiers: SVM, KNN, decision tree, MLP, naive Bayes, and k-means clustering. As depicted in Table 2, the highest precision values for all classifiers corresponded to the biting state with respective values of 99.21%, 97.91%, 97.04%, 100%, 96.17%, and 93.61% for SVM, KNN, DT, MLP, Naive Bayes and K-means Clustering, respectively. Compared to other states, such as chew, chew-bite, and sort, the accuracy of detecting the biting state was elevated. This can be attributed to its higher sound intensity level and distinctiveness, which is evident across all classifiers. In contrast, the lowest precision values for all classifiers were associated with the chewing-biting state, with respective values of 89.07%, 86.12%, 82.14%, 86.21%, 84.12%, and 79.87%. This can be attributed to the interference caused by the overlapping sound intensity of signals generated by jaw movements in both chewing and chewing-biting states, presenting a significant challenge for all classifiers. Based on the classification results, the most effective classifiers for biting, chewing, chewing-biting, and sorting states were identified as MLP, KNN, SVM, and SVM, respectively, with respective values of 100%, 97.91%, 89.07%, and 97.73% Conversely, the least successful classifiers for these states were the first three modes k-means, and Bayes, with respective values of 93.61%, 91.53%, 79.87%, and 92.79%.

A decrease in sensitivity was observed across all classifiers due to the misclassification of chewing-biting and sorting data within the biting category and the misclassification of chewing-biting data within the chewing category. Although MLP and KNN classifiers performed better in classifying biting and chewing states than the SVM classifier, SVM outperformed both in classifying chewing-biting and sorting. Overall, the SVM classifier showed higher accuracy and sensitivity in classifying all four mentioned states than other classifiers. The highest average F1 score (95.66%) was obtained using the SVM algorithm, which consistently exhibited higher F1 scores than other classifiers and clustering methods across nearly all instances of jaw movements.

**Figure 4:**
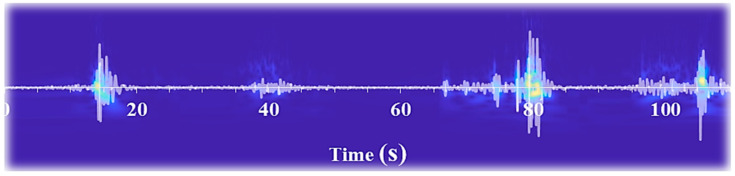
A comparison of the sound signal and the corresponding image generated from it.

The images derived from the transformation of bovine mandibular movements during feeding were scrutinized for conformity concerning the temporal intervals of the events. To facilitate a comparison between the timing of each action, including biting, mastication, chewing-biting, and sorting, in both audio signals and images, the audio signals were superimposed onto the images and subjected to visual analysis. Figure 4 exemplifies this comparison, highlighting that the timing of each action coincides with abrupt fluctuations in image intensity within the corresponding time intervals. Furthermore, it is anticipated that the energy level of the image also increases at the peak points of the sound, which was precisely observed. Thus, by employing intelligent image thresholding, all events arising from the animal’s mandibular movements during feeding can be ascertained regarding their timing and range. In this regard, the classifiers mentioned above can be employed.

**Figure 5:**
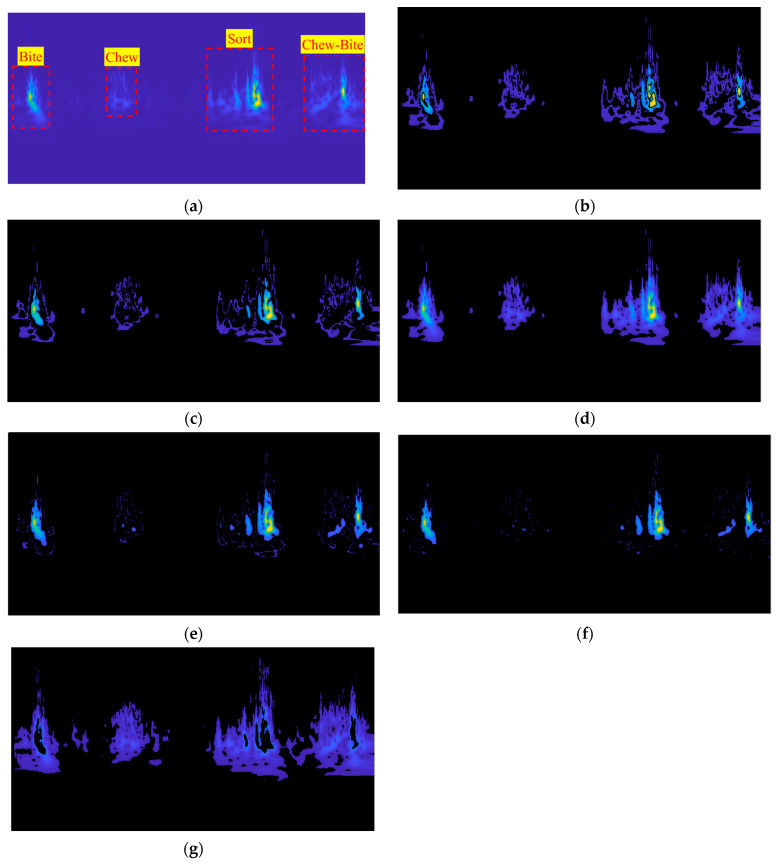
The results demonstrate the pixel classification of each event (**a**) using the classification algorithms SVM (**b**), KNN (**c**), MLP (**d**), decision tree (**e**), naive Bayes (**f**), and k-means clustering (**g**).

Figure 5a illustrates the transformation of an audio signal resulting from the mandibular movements of a cow during the feeding process. As observed in the image, the variation in sound intensity creates a color spectrum ranging from magenta to yellow, where magenta represents the lowest sound intensity, and yellow indicates the highest sound intensity. Since magenta represents the lower sound produced from the jaw movement of the animal, regions consisting solely of magenta color can be associated with chewing. Additionally, the yellow points denote pronounced impacts between the upper and lower jaws or mandibular movements of the cow while selecting the best nutritional content in the food (a process known as sorting).

In light of this observation, three alternative interpretations may be proposed. The first interpretation refers to the points created by the proximity of yellow regions, which exhibit markedly low magenta color intensity. This phenomenon may be attributed to the biting process, as there are strong collisions between the upper and lower jaws during this action, generating a high-intensity sound. In this state, chewing is absent, as evidenced by the minimal presence of magenta regions. The second interpretation concerns the appearance of yellow-colored points close to magenta areas. When the animal commences sorting food for desirable nutritional content using its jaw, it produces a high-intensity sound. The number of yellow regions in the image is anticipated to be larger than the chewing-biting state. The third interpretation involves a higher amalgamation of magenta and yellow colors, representing the concurrent occurrence of biting and mastication. In this state, the magenta combined with yellow is higher than in all states, as mentioned earlier.

Figure 5b–g illustrates the outcomes of thresholding utilizing the mentioned classifiers. The images show that the MLP classifier has demonstrated superior performance in pixel thresholding, whereas k-means clustering has exhibited inferior performance. Upon further evaluation of the classifiers, it may be asserted that their performance has been satisfactory. However, detecting chewing pixels and their subsequent thresholding has presented difficulties and challenges for both the naive Bayes and decision tree classifiers.

## 4. Discussion

The present study successfully classified cow mastication sounds into four classes using vibration analysis. The study employed a rigorous approach to feature selection, using a GA to identify the most pertinent and significant features from a large set of available features. The outcome of the feature selection algorithm yielded five features (entropy, energy, homogeneity, contrast, and moment 2), which were determined to impact classification performance. These selected features were then input into six classifiers: SVM, KNN, decision tree, MLP, naive Bayes, and k-means clustering. The results showed that the highest precision values for all classifiers corresponded to the biting state, and the lowest precision values were associated with the chewing-biting state. The most effective classifiers for biting, chewing, chewing-biting, and sorting states were identified as MLP, KNN, SVM, and SVM, respectively. The results of the study showed that the SVM and KNN classifiers achieved the highest classification precision values of 95.9% and 94.4%, respectively. These results are consistent with the known ability of both classifiers to effectively handle complex and high-dimensional datasets. The superior performance of the SVM classifier can be attributed to its ability to find a hyperplane that maximizes the margin between the two classes, thereby improving the generalization performance of the classifier. Similarly, the high precision of the KNN classifier can be explained by its ability to find the k nearest neighbors to a given data point and assign it to the class that is most common among those neighbors. The decision tree and MLP classifiers also performed well, with precisions of 92.8% and 94.1%, respectively, which can be attributed to their ability to learn complex relationships between features. Unexpectedly, the naive Bayes classifier had a lower precision value compared to the other classifiers. This could be due to its assumption of feature independence, which may not hold true in real-world datasets. The k-means also had a relatively low precision value of 89.6%, which could be due to the fact that it is an unsupervised learning algorithm and may not be suitable for this classification task. Furthermore, the study has faced challenges in accurately classifying the chewing-biting state due to the overlapping sound intensity of signals generated by jaw movements in both chewing and chewing-biting states. This may have presented a significant challenge for all classifiers.

Additionally, the study may have been limited by the number and type of textural features extracted from the images. It is possible that other textural features or feature extraction methods might have improved classification performance. This result is consistent with several previous studies investigating various techniques and methods for accurately detecting and classifying jaw movements in feeding animals. Clapham et al. [58] employed parameters including frequency, intensity, duration, and time between events, calculated from sound segments ranging from 1 to 5 min, to detect and analyze bites. They achieved an overall behavior classification accuracy of 94% and could differentiate between bites and chews with a 94% accuracy using a discriminate function. Navon et al. [59] applied a machine learning technique to 10 min grazing session recordings captured by a camera and achieved a 94% accuracy in jaw movement detection. This accuracy was compared to the analysis of sounds by a trained operator. In a different model, the acoustic spectrum characteristics, specifically the energy produced in decibels by each sound, were used to estimate the sequences of bites, chews, and chew-bites, representing three types of jaw movements. These hidden states are not directly observable but can be inferred through the model [60]. The accuracy of correctly classifying these three jaw movement types ranged from 61% to 99% and was found to be influenced by factors such as pasture type and grass height [61].

Ungar et al. [62] employed discriminant analysis, logistic regression, and neural networks as classification methods and reported accuracy rates ranging from 67% to 82%, 87%, and 25% to 90%, respectively, in correctly classifying jaw stats. Giovanetti et al. [63] successfully used stepwise discriminant analysis (SDA), canonical discriminant analysis (CDA), and discriminant analysis (DA) to automatically evaluate specific behaviors using a triaxial accelerometer (such as the biting activity of dairy sheep in grazing environments). The accuracy of predicting grazing, ruminating, and resting behaviors varied between 89% and 95%, resulting in an overall accuracy of 93%. Chelotti et al. [64] devised a pattern recognition method to categorize jaw movements in grazing cattle based on acoustic signals, yielding a recognition rate of 90% even under noisy conditions.

## 5. Limitations

The study has faced several technical and technological limitations that could have impacted its ability to classify cow mastication sounds accurately. One potential limitation is the quality and accuracy of the recording equipment used to capture the mastication sounds. Furthermore, the recording equipment’s sensitivity to background noise or interference could have impacted the quality of the recorded sounds and made it more challenging to classify mastication behavior accurately. Another limitation is the location of the microphone used to record the sounds. If the microphone was not positioned correctly or was not sensitive enough to accurately capture the sounds, this affected the quality of the recorded data.

Additionally, using a single microphone may not provide a complete picture of the animal’s chewing behavior, as sounds may vary depending on the location and orientation of the microphone. This will be examined in future work. In summary, several technical and technological factors could have influenced the study’s ability to classify cow mastication sounds using vibration analysis accurately. Careful consideration of these factors is important when designing and conducting such studies to ensure accurate and reliable results.

## 6. Conclusions

The proposed approach has successfully classified jaw movements into four distinct categories under tall and short particle sizes conditions in wheat straw and Alfalfa hay feeding. The study has demonstrated that texture features extracted from sound images can be a practical approach for classifying jaw movements in livestock feeding. The results of this study can help develop automated systems for monitoring livestock feeding behavior and improving feed efficiency. The most effective classifiers for each state have been identified, which can be used as a guideline for future studies in this field. Gaining insight into the feeding behavior of dairy cows can help in understanding their feeding requirements and behaviors. Overall, this research has contributed to the advancement of precision livestock farming and has the potential to benefit both livestock producers and researchers.

## Figures and Tables

**Table 1:** Parameters used in genetic algorithms (GA).

GA Parameter	Value
Population size	100
Genome Length	100
Population type	Bit strings
Fitness Function	KNN-Based Classification Error
Number of generations	300
Crossover	Arithmetic Crossover
Crossover Probability	0.8
Mutation	Uniform Mutation
Mutation Probability	0.1
Selection scheme	Tournament of size 2
Elite Count	2

**Table 2:** Classification results of four cow jaw movements with six famous classifications.

Classifier	Jaw Movements	Precision	Recall	F1 Score	Total		
					Precision	Recall	F1 Score
SVM	Bite	99.21	93.59	96.32	95.90	95.76	95.66
	Chew	96.73	93.79	95.24			
	Chew-Bite	89.07	96.47	92.62			
	Sort	97.73	99.19	98.45			
KNN	Bite	98.12	89.32	93.51	94.48	94.71	94.45
	Chew	97.91	93.90	95.86			
	Chew-Bite	86.12	96.63	91.07			
	Sort	95.79	98.99	97.36			
Decision Tree	Bite	97.63	88.73	92.97	92.80	93.11	92.73
	Chew	97.07	89.74	93.26			
	Chew-Bite	82.14	95.63	88.37			
	Sort	94.37	98.35	96.32			
MLP	Bite	100	92.42	96.06	94.18	93.25	93.63
	Chew	94.80	90.57	92.64			
	Chew-Bite	86.21	94.31	90.08			
	Sort	95.73	95.73	95.73			
Naive Bayes	Bite	96.17	86.87	91.28	91.62	91.82	91.61
	Chew	93.42	91.61	92.51			
	Chew-Bite	84.12	90.97	87.41			
	Sort	92.79	97.86	95.26			
K-means Clustering	Bite	93.61	88.44	90.95	89.62	89.58	89.54
	Chew	91.53	89.05	90.27			
	Chew-Bite	79.87	87.24	83.39			
	Sort	93.46	93.61	93.53			

## Data Availability

Data will be available upon request to the corresponding author.

## References

[1] Nleya S.M., Ndlovu S. (2021). Smart dairy farming overview: Innovation, algorithms and challenges. Smart Agriculture Automation Using Advanced Technologies: Data Analytics and Machine Learning, Cloud Architecture, Automation and IoT.

[2] Bach A., Calsamiglia S. (2019). Feeding behavior and efficiency in dairy cows. Anim. Front..

[3] Lokhorst C., De Mol R.M., Kamphuis C. (2019). Invited review: Big Data in precision dairy farming. Animal.

[4] Akbar M.O., Shahbaz Khan M.S., Ali M.J., Hussain A., Qaiser G., Pasha M., Akhtar N. (2020). IoT for development of smart dairy farming. J. Food Qual..

[5] Bewley J.M., Schutz M.M., Lago A. (2018). Precision dairy farming: A new approach to managing dairy cows for improved productivity and sustainability. J. Dairy Sci..

[6] Leliveld L.M., Provolo G. (2020). A review of welfare indicators of indoor-housed dairy cow as a basis for integrated automatic welfare assessment systems. Animals.

[7] Simitzis P., Tzanidakis C., Tzamaloukas O., Sossidou E. (2021). Contribution of Precision Livestock Farming systems to the improvement of welfare status and productivity of dairy animals. Dairy.

[8] Bewley J. (2017). Exploring the potential of precision dairy tools. Proceedings of the 8th Nordic Feed Science Conference.

[9] Braun U., Zürcher S., Hässig M. (2015). Evaluation of eating and rumination behaviour in 300 cows of three different breeds using a noseband pressure sensor. BMC Vet. Res..

[10] Stangaferro M.L., Wijma R., Caixeta L.S., Al-Abri M.A., Giordano J.O. (2016). Use of rumination and activity monitoring for the identification of dairy cows with health disorders: Part I. Metabolic and digestive disorders. J. Dairy Sci..

[11] Reith S., Hoy S. (2018). Behavioral signs of estrus and the potential of fully automated systems for detection of estrus in dairy cattle. Animal.

[12] Džermeikaitė K., Bačėninaitė D., Antanaitis R. (2023). Innovations in Cattle Farming: Application of Innovative Technologies and Sensors in the Diagnosis of Diseases. Animals.

[13] Zehner N., Umstätter C., Niederhauser J.J., Schick M. (2017). System specification and validation of a noseband pressure sensor for measurement of ruminating and eating behavior in stable-fed cows. Comput. Electron. Agric..

[14] Michie C., Andonovic I., Davison C., Hamilton A., Tachtatzis C., Jonsson N., Gilroy M. (2020). The Internet of Things enhancing animal welfare and farm operational efficiency. J. Dairy Res..

[15] Tzanidakis C., Tzamaloukas O., Simitzis P., Panagakis P. (2023). Precision livestock farming applications (PLF) for grazing animals. Agriculture.

[16] Shorten P.R. (2023). Acoustic sensors for detecting cow behaviour. Smart Agric. Technol..

[17] Wang J., Si Y., Wang J., Li X., Zhao K., Liu B., Zhou Y. (2023). Discrimination strategy using machine learning technique for oestrus detection in dairy cows by a dual-channel-based acoustic tag. Comput. Electron. Agric..

[18] Zhang T., Wang J. (2020). Design and Implementation of Cow Chewing Behavior Recognition Based on Sound Sensor. Data Processing Techniques and Applications for Cyber-Physical Systems (DPTA 2019).

[19] Pandeya Y.R., Bhattarai B., Afzaal U., Kim J.B., Lee J. (2022). A monophonic cow sound annotation tool using a semi-automatic method on audio/video data. Livest. Sci..

[20] Galli J.R., Cangiano C.A., Pece M.A., Larripa M.J., Milone D.H., Utsumi S.A., Laca E.A. (2018). Monitoring and assessment of ingestive chewing sounds for prediction of herbage intake rate in grazing cattle. Animal.

[21] Jung D.H., Kim N.Y., Moon S.H., Jhin C., Kim H.J., Yang J.S., Park S.H. (2021). Deep learning-based cattle vocal classification model and real-time livestock monitoring system with noise filtering. Animals.

[22] Martínez Rau L., Chelotti J.O., Vanrell S.R., Giovanini L.L. Developments on real-time monitoring of grazing cattle feeding behavior using sound. Proceedings of the 2020 IEEE International Conference on Industrial Technology (ICIT).

[23] Mansbridge N., Mitsch J., Bollard N., Ellis K., Miguel-Pacheco G.G., Dottorini T., Kaler J. (2018). Feature Selection and Comparison of Machine Learning Algorithms in Classification of Grazing and Rumination Behaviour in Sheep. Sensors.

[24] Zhao K., Zha Z., Li H., Wu J. (2021). Early detection of moldy apple core based on time-frequency images of vibro-acoustic signals. Postharvest Biol. Technol..

[25] Haralick R.M., Shanmugam K., Dinstein I.H. (1973). Textural features for image classification. IEEE Trans. Syst. Man Cybern..

[26] Kumaran T.S. (2018). Coin recognition using texture feature based on SPLM and SGLDM algorithm. AIP Conference Proceedings.

[27] Raghavendra U., Acharya U.R., Gudigar A., Tan J.H., Fujita H., Hagiwara Y., Ng K.H. (2017). Fusion of spatial gray level dependency and fractal texture features for the characterization of thyroid lesions. Ultrasonics.

[28] Subashini P. (2013). Texture feature extraction of infrared river ice images using second-order spatial statistics. Int. J. Comput. Inf. Eng..

[29] Abadi A.M., Wutsqa D.U., Ningsih N. (2021). Construction of fuzzy radial basis function neural network model for diagnosing prostate cancer. TELKOMNIKA (Telecommun. Comput. Electron. Control.).

[30] Boudraa S., Melouah A., Merouani H.F. (2020). Improving mass discrimination in mammogram-CAD system using texture information and super-resolution reconstruction. Evol. Syst..

[31] Taherdangkoo M., Paziresh M., Yazdi M., Bagheri M.H. (2012). An efficient algorithm for function optimization: Modified stem cells algorithm. Cent. Eur. J. Eng..

[32] Zhang J., Chung H., Lo W.L. (2007). Clustering-Based Adaptive Crossover and Mutation Probabilities for Genetic Algorithms. IEEE Trans. Evol. Comput..

[33] Tian J., Hu Q., Ma X., Ha M. (2012). An Improved KPCA/GA-SVM Classication Model for Plant Leaf Disease Recognition. J. Comput. Inf. Syst..

[34] Akbari R., Ziarati K. (2011). A multilevel evolutionary algorithm for optimizing numerical functions. Int. J. Ind. Eng. Comput..

[35] Babatunde O.H., Armstrong L., Leng J., Diepeveen D. (2014). Zernike moments and genetic algorithm: Tutorial and application. Br. J. Math. Comput. Sci..

[36] Kotsiantis S.B. (2007). Supervised machine learning: A review of classification techniques. Informatica.

[37] Novakovic J., Strbac P., Bulatović D. (2011). Toward optimal feature selection using ranking methods and classification algorithms. Yugosl. J. Oper. Res..

[38] Chatcharaporn K., Kittidachanupap N., Kerdprasop K. Comparison of feature selection and classification algorithms for restaurant dataset classification. Proceedings of the 11th Conference on Latest Advances in Systems Science & Computational Intelligence.

[39] Vapnik V.N. (1998). The Nature of Statistical Learning Theory.

[40] Platt J. (1998). Sequential Minimal Optimization: A Fast Algorithm for Training Support Vector Machines.

[41] Liang X., Zhu L., Huang D.S. (2017). Multi-task ranking SVM for image cosegmentation. Neurocomputing.

[42] Cervantes J., García Lamont F., López-Chau A., Rodríguez Mazahua L., Sergio Ruíz J. (2015). Data selection based on decision tree for SVM classification on large data sets. Appl. Soft Comput. J..

[43] Naik V.A., Desai A.A. Online handwritten gujarati character recognition using SVM, MLP, and K-NN. Proceedings of the 2017 8th International Conference on Computing, Communication and Networking Technologies (ICCCNT).

[44] Raheja J.L., Mishra A., Chaudhary A. (2016). Indian sign language recognition using SVM. Pattern Recogn. Image Anal..

[45] Yeturu K. (2020). Machine learning algorithms, applications, and practices in data science. Handbook of Statistics.

[46] Trabelsi A., Elouedi Z., Lefevre E. (2018). Decision tree classifiers for evidential attribute values and class labels. Fuzzy Sets Syst..

[47] Fratello M., Tagliaferri R. (2019). Decision trees and random forests. Encyclopedia of Bioinformatics and Computational Biology.

[48] Huang D.S., Du J.X. (2008). A constructive hybrid structure optimization methodology for radial basis probabilistic neural networks. IEEE Trans. Neural Netw..

[49] Wang X.F., Huang D.S. (2008). A novel multi-layer level set method for image segmentation. J. Univers. Comput. Sci..

[50] Zhang M., Qu H., Xie X., Kurths J. (2017). Supervised learning in spiking neural networks with noise-threshold. Neurocomputing.

[51] Huang L., Cui Y., Zhang D., Wu S. (2011). Impact of noise structure and network topology on tracking speed of neural networks. Neural Netw..

[52] Li Y., Wu H. (2012). A clustering method based on K-means algorithm. Phys. Procedia.

[53] Estivill-Castro V., Yang J. (2004). Fast and robust general purpose clustering algorithms. Data Min. Knowl. Discov..

[54] Altman N.S. (1992). An introduction to kernel and nearest-neighbor nonparametric regression. Am. Statist..

[55] Zhang Y., Cao G., Wang B., Li X. (2019). A novel ensemble method for k-nearest neighbor. Pattern Recogn..

[56] Gebejes A., Huertas R. (2013). Texture characterization based on grey-level co-occurrence matrix. Databases.

[57] Yu S.N., Huang Y.K. (2010). Detection of microcalcifications in digital mammograms using combined model-based and statistical textural features. Expert Syst. Appl..

[58] Clapham W.M., Fedders J.M., Beeman K., Neel J.P. (2011). Acoustic monitoring system to quantify ingestive behavior of free-grazing cattle. Comput. Electron. Agric..

[59] Navon S., Mizrach A., Hetzroni A., Ungar E.D. (2013). Automatic recognition of jaw movements in free-ranging cattle, goats and sheep, using acoustic monitoring. Biosyst. Eng..

[60] Ungar E.D., Rutter S.M. (2006). Classifying cattle jaw movements: Comparing IGER behavior recorder and acoustic techniques. Appl. Anim. Behav. Sci..

[61] Milone D.H., Rufiner H.L., Galli J.R., Laca E.A., Cangiano C.A. (2009). Computational method for segmentation and classification of ingestive sounds in sheep. Comput. Electron. Agric..

[62] Ungar E.D., Nevo Y., Baram H., Arieli A. (2018). Evaluation of the IceTag leg sensor and its derivative models to predict behaviour, using beef cattle on rangeland. J. Neurosci. Methods.

[63] Giovanetti V., Decandia M., Molle G., Acciaro M., Mameli M., Cabiddu A., Dimauro C. (2017). Automatic classification system for grazing, ruminating and resting behaviour of dairy sheep using a tri-axial accelerometer. Livest. Sci..

[64] Chelotti J.O., Vanrell S.R., Galli J.R., Giovanini L.L., Rufiner H.L. (2018). A pattern recognition approach for detecting and classifying jaw movements in grazing cattle. Comput. Electron. Agric..

